# Clusters of alcohol and drug use and other health-risk behaviors among Thai secondary school students: a latent class analysis

**DOI:** 10.1186/s12889-018-6205-z

**Published:** 2018-11-20

**Authors:** Sawitri Assanangkornchai, Jing Li, Edward McNeil, Darika Saingam

**Affiliations:** 1 Epidemiology Unit, Faculty of Medicine, Prince of Songkla University, Hat Yai, Songkhla, Thailand; 2 Kunming Medical University, Kunming, Yunnan Province China

**Keywords:** Latent class analysis, Health-risk behavior, Secondary school students, Alcohol, Tobacco and drug use

## Abstract

**Background:**

Alcohol and drug use and other health-risk behaviors tend to cluster together among adolescents and contribute a large amount of harm to both themselves and to others. This paper aims to characterize secondary school students based on their clusters of health-risk behaviors and identify factors determining class membership to these behavior-clusters.

**Methods:**

Data from a national school survey was used to identify clusters of alcohol and drug use and other health-risk behaviors among secondary school students aged 12–15 years using a latent class regression model. A multinomial logistic regression model was used to identify predictors of the cluster membership.

**Results:**

A total of 25,566 students were included in the analysis, of which 88% were classified as having low-risk behaviors reporting only moderate alcohol use; 11% as having moderate-risk behaviors, such as driving under the influence of alcohol, fighting, carrying a weapon, and alcohol and tobacco use; and 0.6% as having high-risk behaviors, such as use of illicit drugs, particularly kratom and cannabis. Males, older students, those with a poor school performance, not living with parents, drug use by family members and peers, and having a low level of perceived disdain from their friends if they used drugs were significant risk factors for being in the moderate- and high-risk behavior classes.

**Conclusions:**

Alcohol, tobacco and drug use, as well as other health-risk behaviors such as fighting, are clustered in Thai secondary school students. This result highlights the importance of comprehensive prevention and education strategies, particularly for moderate to high-risk groups.

## Background

Health-risk behaviors consist of many categories: behaviors that contribute to unintentional injuries and violence, sexual behaviors that contribute to unintended pregnancy and sexually transmitted diseases, alcohol, tobacco, and other drug use, unhealthy dietary behaviors and inadequate physical activities [1]. These behaviors are common among adolescents and contribute to a large amount of harm to both themselves and to others with whom they may come into contact [2–4].

Generally, alcohol and drug use and other health-risk behaviors tend to be clustered together [5], especially during adolescence when significant physical, sexual and psychological development occurs [3, 6–8]. Examples include the co-occurrence of alcohol and tobacco use, early sexual debut, and bullying [2], a clustering of high-fat diet, irregular physical activity and sedentary behaviors [9] and an inter-relationship between alcohol use, marihuana use, sexual risk behaviors and delinquency [10]. Moreover, depressive symptoms were found to occur together with risk behaviors among adolescents and young adults [6]. Such clustering has important implications for research and practice since some behaviors share a similar feature, and as a consequence, changing one behavior affects the prevalence of another [10]. Understanding how young adolescents’ behaviors cluster across individuals in an education setting could help inform how best to adapt prevention programs to more successfully serve different subgroups. Interventions that simultaneously tackled health behaviors which are clustered have been shown to be more effective and less costly [11].

Methods such as cluster analysis or latent class analysis (LCA), the latter aiming to classify people into mutually exclusive groups with which they share similar characteristics, have been commonly used. Examples include studies of health-related behaviors with relevant demographic factors and health-related outcomes [7], polysubstance use [12] and profiles of risk behaviors and mental health concerns among adolescents [13], clustering of smoking, poor nutrition, excess alcohol consumption, and physical inactivity among adults [8]. LCA identifies unobservable subgroups within a population, allowing a better understanding of the impact of an exposure to patterns of multiple risks as well as the antecedents and consequences of complex behaviors so that interventions can be tailored to target the subgroups that will benefit most [14]. Tomczyk and colleagues reported a systematic review of studies using latent class analysis to examine classes of polysubstance use in adolescents (10–19 years), and to describe predictors of polysubstance use. They found that predictors of polysubstance use classes included higher age, higher parental and peer substance use, and poor academic performance. They concluded that latent classes could deliver solid information on polysubstance use in adolescents and help to illustrate differential effects and special groups in prevention and treatment that depend on the actual substance use pattern [12].

Risk behaviors in adolescents are multi-factorial and are associated with individual factors, family characteristics, and social and environmental conditions [15, 16]. Studies have found that alcohol, tobacco and drug use are influenced by factors such as family relationships, peer groups, and school environment [3, 16–18]. Based on Family Interaction theory, parenting styles, familial dysfunction, parent-child relations, and parental substance use could contribute to the intergenerational transmission of substance use disorders on children [19–21]. Apart from the family, which provides the first context for infants and very young children, as children grow, the wider environment at school and social interaction with friends take on a larger role. The norms created by peers have increasing influence compared to family members on the adolescent’s behavioral development as the individual gets older [22]. Moreover, differences in gender, age, and the family’s socio-economic status were seen for risk-taking behaviors among adolescents, such as alcohol and cannabis consumption, bullying and fighting, early sexual debut, and smoking [2, 23]. Understanding the underlying determinants of health-risk behaviors in young people is essential if interventions are to be developed to prevent these behaviors.

As an effect of globalization, Thailand, in recent decades, has seen tremendous changes in people’s lifestyles, family and social relationships and values. Adolescents, even those aged in their early teens, are more independent of their family and as a consequence have become more engaged in health-risk behaviors, including alcohol, tobacco and drug use, premature and unsafe sex, and violent behaviors such as carrying a weapon or fighting [24–26]. Our previous school surveys in southern Thailand in 2002–2004 found an increasing trend of substance use among adolescents [27]. In addition, a high prevalence of risk behaviors in the 30 days prior to the survey, such as riding a motorcycle without wearing a helmet (72.7%) or after drinking alcohol (13.1%), carrying a weapon (7.6%) and fighting that involved a physical harm (14.0%) was found in our previous school survey in 2005 [26].

Most previous studies in Thailand have focused on single risk behaviors. This is the first report in Thailand aiming to characterize adolescents based on clusters of their health-risk behaviors and identify individual, family and school environmental factors determining membership of the risk behavior clusters.

## Methods

### Sample

The 2009 national school survey was conducted among students in levels 1, 3, and 5 of high schools, which are equivalent to years 7, 9, and 11 of the international education system, and level 2 of vocational schools. A two-stage stratified cluster sample survey was conducted. In Thailand, school administration is divided into three zones in Bangkok, and 12 other educational zones in the rest of the country. In each zone, 40–50% of the provinces were randomly selected, giving 37 provinces throughout the country and three Bangkok zones. In each province, 4–5 schools were randomly selected including one public high school in an urban area, one in a rural area, one private school, one commercial vocational school, and one technical vocational school. Totally 197 schools participated in the study. In each school, 3–5 classes of each level were randomly selected. The sampling technique and data collection method were similar to that described in the national school survey conducted in 2007 [24].

All students in the selected classes were asked to complete a self-administered, voluntary and anonymous questionnaire during their regular class period. Data collection was done between December 2009 and February 2010, the second semester of the school year in Thailand.

In this study, only students from junior high schools (years 7 and 9), representing early teenagers aged between 12 and 15 years, were included. Altogether, 25,566 students from 119 schools were included in this analysis - resulting in a 99% participation rate. About half (54%) were female and most (92%) were Buddhists.

### Manifest variables

Twenty manifest variables representing four categories of health-risk behaviors that contribute to the leading causes of death and disability and common among youths [1] were included in the LCA model. They included 1) behaviors that contribute to unintentional injuries and violence in the past 12 months, 2) behaviors that contribute to intentional injuries in the past 12 months, 3) past-month alcohol use, and 3) past-year drug use.

For past-year drug use, students answered whether or not they had ever used any of the following drugs in the past 12 months: anxiolytics/hypnotics, cannabis, cough syrup (a commonly abused substance by young people in Thailand as it contains codeine), cocaine, crystalline amphetamine, ecstasy, heroin, inhalants, ketamine, kratom (Mitragynine speciosa, Kroth.) or kratom cocktail (a mixture of kratom tea, cough syrup and cola), methamphetamine, opium, and tobacco. Examples of common street names of each drug were included in the question. For anxiolytics/hypnotics and cough syrup, the question specified that the use must have been for non-medicinal purposes.

As alcohol consumption was more common than other drugs and quantity of consumption and episodes of binge drinking were considered important in classifying harmful patterns of alcohol consumption, three variables were used to measure alcohol consumption in the past 30 days of the study. These included (1) current alcohol consumption (defined as drinking at least one standard alcoholic drink, not including 1–2 sips for tasting, at least once in the past 30 days prior to the study); (2) heavy drinking (defined as drinking 2 or more standard drinks during a normal drinking occasion) and (3) frequent binge drinking (defined as having at least one episode of drinking 5 or more drinks in a drinking occasion during the past 4 weeks). Behaviors that contributed to unintentional injuries and violence in the past 12 months included driving a motorcycle or car after drinking alcohol; carrying a weapon; and fighting. Behaviors that contributed to intentional injuries in the past 12 months included suicidal risk, defined as having suicidal thoughts, suicidal plans, or suicide attempts in the past 12 months.

All manifest variables were treated as dichotomous. The questionnaire of the national school surveys in Thailand was derived from questions contained in the US Youth Risk Behavior Surveillance System [1], the U.S. Monitoring the Future Project [28], and the National Household Survey of Substance Use in Thailand [29].

### Predictors of latent class membership

#### Individual factors

Individual factors included age, gender, religion, school class and grade point average (GPA). In Thailand, GPA is categorized into four levels of scores ranging from 0 to 4.0, with 4.0 representing the highest grade and 0 representing the lowest. Other individual factors included living with parents or other adult relatives (compared to living alone or with friends), and attitude towards drug use, measured by asking if the students thought that their friends would have a feeling of disdain towards them if they used alcohol, tobacco, and each drug. Each student’s response was coded as 1 for yes and 0 for no and these were summed giving a total score ranging from 0 to 15 where higher scores indicate a higher feeling of disdain. The median was used as a cut-off score to classify the students’ perceived feeling of disdain by their friends towards their own drug use into either high or low level. Perceived roughness of school was measured with seven questions asking if the students perceived that other students in their school had each of the following behavioral problems: physical assault, drug use, suicide, rape or sexual assault, heavy drinking, group fighting, and interpersonal quarreling. Answers to each question ranged from not at all (0), a little (1), moderate (2) and a lot of problems (3). The total score for perceived roughness of the school was derived from the sum of these scores for all seven questions and cut into high and low levels using the median.

#### Peer and family factors

Peer and family factors included six questions asking if any of the student’s family members or friends currently had alcohol, tobacco or other drug-related problems. Family members listed in the questionnaire included mother, father, step-mother, step-father, grandmother, grandfather, sibling and other close relative.

### Statistical analysis

Percentages with standard errors (SE) of alcohol consumption, drug use, and other health risk behaviors were described using sampling weights adjusted for the two-stage stratified sampling design.

Patterns of health-risk behaviors including alcohol, tobacco, and drug use were produced using a latent class regression model. Students were considered to have significant differences in their prior probabilities of latent class membership in the different regions in Thailand [30]. Therefore, region, of which there are five (central, north, northeast, south and Bangkok and its periphery), was included in the latent class regression model as a covariate.

A series of models with increasing number of classes was constructed to determine the one that offered the best fit to the data. The model parameters included class membership probabilities (or class prevalence estimates) and class-specific symptom endorsement probabilities. A heuristic approach was employed for model assessment using the Akaike Information Criterion (AIC), Bayesian Information Criterion (BIC), sample size adjusted Bayesian Information Criterion (SSABIC) and the maximum log-likelihood value as measures to determine the model fit which best balanced the number of parameters and information from those parameters as well as the parsimony principle, i.e. fewer parameters estimated [31, 32]. Other considerations of fit were the ability to assign meaningful labels to the classes and having no individual class which contained very few students. Relative entropy was calculated based on the equation given by Gilles Celeux and G. Soromenho [33].

After fitting the models, suitable latent class membership was determined. Then, a multinomial logistic regression model was used to identify independent predictors of latent class membership. The best-fitting model was selected based on the minimum AIC. Relative risk ratios (RRR) and corresponding 95% confidence intervals compared to the baseline class of latent class membership were presented.

Data management, latent class regression, and multinomial logistic regression models were conducted using R with the “poLCA” user-contributed package [30].

## Results

### Percentages of health risk behaviors

The majority (60.5%) of students (*n* = 15,470) had no health-risk behaviors during the past year (weighted percent = 59.2%, SE = 0.4%). Table 1 demonstrates that fighting was the most common risk behavior among Thai students accounting for 20.6% (SE = 0.3), followed by alcohol consumption (17.8%; SE = 0.3) and carrying a weapon (14.3%; SE = 0.3). Drugs were not commonly used, the highest prevalence being for tobacco (5.3%), cannabis (1.0%) and cough syrup (0.8%).

**Table 1:** Distribution of drinking, smoking, drug use and other health-risk behaviors among junior high school students in Thailand, 2009

Behavior	n	Percent (SE)^a^
Unintentional injuries/violence (past 12 months)		
Fighting (fig)	5143	20.6 (0.31)
Carrying a weapon (wea)	3553	14.3 (0.27)
Driving after drinking (dui)	1784	7.5 (0.21)
Intentional injuries (past 12 months)		
Suicidal intent/attempts (sui)	2033	8.4 (0.22)
Alcohol use (past 30 days)		
Current drinking (alc)	4107	17.8 (0.30)
Heavy drinking (hvy)	1598	6.9 (0.20)
Binge drinking (bin)	767	3.4 (0.15)
Drug use (past 12 months)		
Tobacco (tob)	1386	5.3 (0.17)
Cannabis (can)	244	1.0 (0.08)
Cough syrup (cou)	209	0.8 (0.07)
Krathom (kra)	183	0.6 (0.06)
Inhalants (inh)	122	0.5 (0.06)
Methamphetamine (met)	121	0.5 (0.05)
Anxiolytics (anx)	100	0.4 (0.05)
Crystalline amphetamine (ice)	80	0.3 (0.05)
Opium (opi)	68	0.3 (0.04)
Ecstasy (ecs)	67	0.3 (0.04)
Heroin (her)	57	0.3 (0.04)
Cocaine (coc)	54	0.2 (0.04)
Ketamine (ket)	53	0.2 (0.04)
Any health-risk behavior	9474	38.4 (0.37)

^a^Percentages and standard errors (SE) are estimated based on sampling weights

### Latent class regression model

Fit indices for the model on the 20 health-risk behavior variables were calculated with results shown in Table 2. All of the criteria (AIC, BIC, SSABIC, and maximum log-likelihood) suggested that the four-class model fit the data best, followed closely by the three-class model. However, the three-class model had the best balance in the number of parameters and the information from those parameters. The entropy of the three-class model was also higher than that of the four-class model (0.82 vs 0.62), indicating more certainty in the separation of classes.

**Table 2:** Fit indices for the latent class regression model with region as a covariate for adolescent health-risk behaviors

	1 class	2 classes	3 classes	4 classes	5 classes
AIC	139,301	116,607	112,834	109,369	112,922
BIC	139,464	116,949	113,355	110,070	113,802
SSABIC	139,400	116,662	112,845	109,337	112,845
Max. LL	−69,630	− 58,262	−56,353	−54,599	−56,353
Entropy	–	0.85	0.82	0.62	–

*AIC* akaike information criterion, *BIC* Bayesian Information Criterion, *SSABIC* Sample Size Adjusted Bayesian Information Criterion, *Max. LL* Maximum Log-likelihood statistic. -: Entropy not computable

**Fig. 1:**
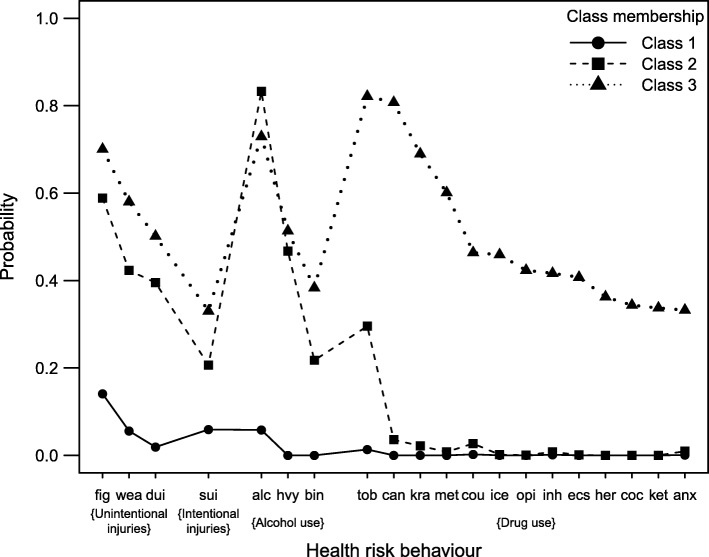
Item-response probabilities of health-risk behaviors for a three-class latent membership model. Health-risk behaviors: Violence/unintentional injuries: fig = Fighting; wea = Carrying a weapon; dui = Driving under the influence of alcohol. Intentional injuries: sui = Suicidal intentions. Alcohol consumption: alc = Drinks alcohol; hvy = Heavy drinking; bin = Binge drinking. Drug use: tob. = Tobacco; can = Cannabis; kra. = Kratom/kratom cocktail; met = Methamphetamine; cou = Cough syrup; ice = Crystalline amphetamine; opi = Opium; inh = Inhalants; ecs = Ecstasy; her = Heroin; coc = Cocaine; ket = Ketamine; anx = Anxiolytics

The three latent classes represented a continuum of levels of different health-risk behaviors. The majority (87.7%) of students were assigned to class 1, which consisted of low probabilities (< 0.2) of alcohol consumption (without binge or heavy drinking), fighting and suicidal behavior. It was labeled as a “low-risk” class with almost no health-risk or drug-related behaviors. About 11.7% of students were assigned to class 2, which was primarily characterized by higher probabilities of fighting, carrying a weapon, driving after drinking alcohol, alcohol consumption and tobacco use. This class was labeled as a “moderate-risk” class due to the students’ low frequency of drug use and high alcohol use and other health-risk behaviors. Finally, a small proportion (0.6%, *n* = 155) of students were allocated to class 3, which had the highest probability of drug use with alcohol consumption and other health risk behaviors. Consequently, this group was labeled as a “high-risk” class. Figure 1 illustrates the item-response probabilities of health-risk behaviors for the three-class latent membership model grouped by the four health-risk behaviour categories and then sorted by the probability of membership to class 3.

### Differences between classes

Table 3 presents a comparison of socio-demographic characteristics and other factors between the three classes. The majority of students in class 1 were female and aged less than 14 years, studying in high school grade 1 (year 7), had higher scores of opposition to drug use and had no peers with drug use problems. In contrast, the majority of students in classes 2 and 3 were male, aged 15 years, were studying in grade 3 (year 9), had a lower level of perceived feeling of disdain by their friends towards their own drug use, and had peers with drug use problems.

**Table 3:** Distribution of demographic variables by latent class

	Class 1 (*n* = 22,417)	Class 2 (*n* = 2994)	Class 3 (*n* = 155)
Gender			
Male	9713 (43.3)	1924 (64.3)	139 (89.7)
Female	12,704 (56.7)	1070 (35.7)	16 (10.3)
Age (years)			
12	2484 (11.1)	136 (4.5)	4 (2.6)
13	9341 (41.7)	695 (23.2)	30 (19.4)
14	3470 (15.5)	573 (19.1)	25 (16.1)
15	7122 (31.8)	1590 (53.1)	96 (61.9)
Religion			
Buddhist	20,644 (92.1)	2849 (95.2)	125 (80.6)
Muslim/Christian	1773 (7.9)	145 (4.8)	30 (19.4)
High school grade			
Grade 1	12,930 (56.8)	787 (29.8)	37 (24.5)
Grade 3	9844 (43.2)	1854 (70.2)	114 (75.5)
GPA			
0–2.0	1892 (8.4)	570 (19)	56 (36.1)
2.1–3.0	11,323 (50.5)	1700 (56.8)	70 (45.2)
3.1–4.0	9202 (41.0)	724 (24.2)	29 (18.7)
Living status			
Alone or with friends/relatives	4260 (19)	634 (21.2)	33 (21.3)
With parents	18,157 (81)	2360 (78.8)	122 (78.7)
School type			
Public	15,423 (68.8)	1902 (63.5)	99 (63.9)
Private	6994 (31.2)	1092 (36.5)	56 (36.1)
Disdain from friends if use drugs			
Low level	11,109 (49.6)	2234 (74.6)	138 (89.0)
High level	11,308 (50.4)	760 (25.4)	17 (11.0)
Perceived roughness^a^ of school			
Low level	15,729 (70.2)	1910 (63.8)	80 (51.6)
High level	6688 (29.8)	1084 (36.2)	75 (48.4)
Alcohol use problem of family members			
No	14,991 (66.9)	1662 (55.5)	102 (65.8)
Yes	7426 (33.1)	1332 (44.5)	53 (34.2)
Tobacco use problem of family members			
No	16,607 (74.1)	1938 (64.7)	98 (63.2)
Yes	5810 (25.9)	1056 (35.3)	57 (36.8)
Drug use problem of family members			
No	22,095 (98.6)	2853 (95.3)	129 (83.2)
Yes	322 (1.4)	141 (4.7)	26 (16.8)
Alcohol use problem of peers			
No	15,354 (68.5)	1199 (40.0)	47 (30.3)
Yes	7063 (31.5)	1795 (60.0)	108 (69.7)
Tobacco use problem of peers			
No	9324 (41.6)	469 (15.7)	14 (9)
Yes	13,093 (58.4)	2525 (84.3)	141 (91)
Drug use problem of peers			
No	16,638 (74.2)	1546 (51.6)	39 (25.2)
Yes	5779 (25.8)	1448 (48.4)	116 (74.8)

*GPA* grade point average

^a^Includes seven items: physical assault, drug use, suicide, rape or sexual assault, heavy drinking, group fighting and interpersonal quarrelling

### Multinomial logistic regression

Table 4 shows the results of the multinomial logistic regression analysis using the low-risk class as the baseline outcome group. Males, those aged 15 years and having a lower GPA were more likely to be in the moderate-risk and high-risk behavior classes. Although Muslims were less likely to be in the moderate-class compared to Buddhists, they were more likely to be in the high-risk class. Those who perceived that their friends had less disdain for their own drug use behaviors were more likely to be in the moderate- and high-risk classes.

**Table 4:** Relative risk ratios (RRR) and 95% confidence intervals (CI) with low-risk behavior class as the baseline outcome group

	Moderate-risk		High-risk	
	RRR	95% CI	RRR	95% CI
Male gender	2.09	(1.91–2.28)	10.3	(6.02–17.8)
Age, years (Ref: 12)				
13	1.24	(1.02–1.50)	1.54	(0.53–4.45)
14	2.31	(1.89–2.83)	2.52	(0.86–7.41)
15	2.93	(2.42–3.54)	4.62	(1.67–12.8)
Religion (Ref: Buddhist)				
Muslim/Christian	0.68	(0.57–0.82)	2.96	(1.88–4.67)
GPA (Ref: 3.1–4.0)				
2.1–3.0	1.45	(1.31–1.60)	1.27	(0.81–2.00)
≤ 2.0	1.97	(1.72–2.25)	3.08	(1.89–5.01)
Lives with parents	0.85	(0.77–0.94)	0.85	(0.57–1.28)
Private school (vs public)	1.44	(1.31–1.59)	1.57	(1.11–2.23)
Alcohol use by family members	1.32	(1.19–1.46)	0.70	(0.44–1.11)
Tobacco use by family members	1.28	(1.15–1.43)	1.67	(1.06–2.64)
Drug use by family members	1.98	(1.58–2.48)	8.94	(5.23–15.3)
Alcohol use by peers	1.60	(1.45–1.76)	1.52	(1.01–2.29)
Tobacco use by peers	1.74	(1.55–1.96)	1.52	(0.81–2.84)
Drug use by peers	1.30	(1.18–1.43)	3.45	(2.25–5.29)
Disdain from friends if use drugs (low vs. high level)	2.17	(1.98–2.37)	5.26	(3.14–8.81)
Perceived roughness^a^of school (high vs. low level)	1.21	(1.11–1.32)	2.02	(1.44–2.83)

*GPA* grade point average

^a^Includes seven items: physical assault, drug use, suicide, rape or sexual assault, heavy drinking, group fighting and interpersonal quarrelling

Compared with students in the low-risk class, those whose family members had tobacco use problems were 1.3 and 1.7 times more likely to be in the moderate- and high-risk classes, respectively, while students whose family members had drug use problems were 2 and 9 times more likely to be in the moderate- and high-risk classes, respectively. Additionally, students whose peers had drug use problems were 1.3 and 3.5 times more likely to be in the moderate- and high-risk classes, respectively, while students who had peers with alcohol use problems were 1.6 and 1.5 times more likely to be in the moderate- and high-risk classes, respectively. Finally, students who had a low level of perceived disdain by their friends if they used drugs were 2.2 and 5.3 times more likely to be in the moderate- and high-risk classes, respectively, while those with a high level of perceived roughness of their own school were 1.2 and 2.0 times more likely to be so.

## Discussion

This study empirically supports the heterogeneity of adolescent health-risk behaviors. Our analyses generated a best-fitting three-class model of adolescent health-risk behaviors in Thai high school students. Class 1 (low-risk behaviors) was characterized by small probabilities of fighting, alcohol and tobacco use without other drug use or other health-risk behaviors while both class 2 (moderate-risk behaviors) and class 3 (high-risk behaviors) were similar in terms of having a higher probability of alcohol and tobacco use and other health-risk behaviors; they could be differentiated by the experience of drug use, which was only present in the high-risk class.

Nearly 90% of students were categorized as having a low level of health-risk behaviors. These students were more likely to be female, younger, and have a high GPA. They also had a smaller percentage of friends who had consumed alcohol, tobacco and other illicit drugs, compared to the other classes. Consistent with research in other countries, this study suggests that most adolescents could go through this transitional period successfully as the highest proportion of adolescents were in this low-risk group [3, 13, 34]. For example, Noel and colleagues found that the majority (80% of a four-class LCA model) of students in secondary schools in New Zealand were ‘healthy’ and did not engage in health-risk behaviors or suffer from emotional health concerns [13] and Ji and colleagues found the highest proportion (52% of a four-class LCA model) of middle school students in rural western China was in the low-risk class with lowest probabilities for all risk behaviors such as binge drinking, self-injurious behaviors, suicide and accidental injury [34]. One explanation of the high proportion of class 1 members in our study may be that these students were still young with about 50% being 13 years and under and studying in year 7 so they had not experienced those risk behaviors yet.

Although a small number of students in our study were in the high-risk class (155 students, 0.6%), they were engaging in multiple risk behaviors, including drug use, especially cannabis, kratom or kratom cocktail, and methamphetamine. Over 75% of these students were in year 9 and aged 14 or 15 years, which, in fact, seems to be too young for such risk behaviors. This warrants concerns of school personnel and parents of the possible harm to the students’ health and social wellbeing. Of specific to Thai adolescents was the high probability of kratom or kratom cocktail use among this group as this drug is highly available in Thailand and perceived as a “soft drug” with little or no harmful effect. The kratom cocktail (dubbed “4 × 100”, or “4 times 100”) is a mixture of four ingredients, namely boiled kratom leaves, soft drink such as coca cola, cough syrup, and either coffee or codeine, and is usually chilled with ice [35–37]. It is common among teenagers who drink together in a group or with friends [36, 37]. This issue requires urgent action across multiple sectors, including health, education and social services for screening and early intervention of these high-risk students.

Among the moderate-risk class students, the probability of alcohol consumption was higher than that in the high-risk class and probability of heavy drinking was similar, although frequent binge drinking was lower. Additionally, the probabilities of tobacco smoking, behaviors related to violence or unintentional injuries, and suicidal behaviors were high in this group. The clustering of alcohol consumption with these behaviors was similar to other studies [8, 13, 34] and emphasized the consequences of alcohol drinking among adolescents such as risky sexual activities [38], school absenteeism [39], and automobile accidents [38, 40]. Drinking and smoking most often start in adolescence. Therefore, strategies to prevent smoking and drinking initiation are needed to improve students’ health behaviors at an early age.

From this study, numerous factors illustrate the complexity of health risk behaviors among students. Males and those with low school performance had a higher risk of health-risk behaviors at an early age. Students who were exposed to social disadvantages, such as living without parents, or those whose families and peers consumed alcohol, smoked tobacco and used drugs, had a higher chance of engaging in health-risk behaviors. These findings are similar to another study among secondary school students where males were more likely to be in the high-risk behavior group, which included behaviors such as unhealthy diet, physical inactivity, unhealthy internet use, accidental injury, tobacco use, self-injurious behavior and suicide risk [34]. Muslims were found more likely to be in the high-risk class but less likely in the low-risk class. This may be because Muslims (80% of the ‘other’ religion in this study) do not drink alcohol, so they were less likely to be classified into the moderate-risk class, a class which was characterized by alcohol consumption risk behaviors.

After adjusting for age and gender, the strongest predictors of high-risk class were drug use problems among family members (RRR = 8.94), followed by a low perceived level of disdain by friends if a student used drugs (RRR = 5.26), and drug use by peers (RR = 3.45). These results are consistent with other studies in which having drug using family members and friends increased the risk of health-risk behaviors among adolescents [41, 42]. Similarly, other studies have found that smoking and alcohol use by friends increased the risk of alcohol use and other health-risk behaviors by the adolescents [43, 44] while perceived peer norms on drug use were influential on alcohol and cannabis use among early adolescents [45]. This suggests that adolescent health-risk behaviors can be conceptualized as a result of both peer influence and selection processes, with peer influences enhancing risk-taking among adolescents [46] and adolescents selecting friends who engage in similar levels of risk behaviors.

All of the aforementioned findings highlight the need for appropriate statistical methods, such as latent class regression, to fully understand alcohol, tobacco, and drug use and other health-risk behaviors and their determinants. Using single, broad measures of health-risk behaviors would result in young people who frequently drink heavily or occasionally use drugs and may have behavioral problems being categorized with those who have a low alcohol intake and no risk behaviors at all. As alcohol and drug use is linked to adverse consequences, our findings emphasize the need to focus on adolescents with alcohol and drug use and experiences of other health-risk behaviors, especially among those whose family members and peers have drug use problems. Thailand’s rapid economic development in the past few decades and the effects of globalization have created many changes in the society and family lives of Thais. More urbanization is evident, and people are under more pressure to compete in an increasingly rapid daily lifestyle. Children are left behind with less parental monitoring. Adolescents are also exposed to a lot of stresses in their lives including peer-pressure and competition at school. It is therefore not surprising to find that our results of clusters of health-risk behaviors and their predictors are similar to what has been found in other countries. Positive youth development programs [47] have been promoted in Thailand recently where children and adolescents are encouraged to engage in productive activities and enhance their strengths. A recent nation-wide school curriculum initiative called “Study less, know more” was launched in 2017 aiming to reduce classroom study time and increase out-of-classroom skills development activities for junior high school students (http://www.moe.go.th/websm/2015/sep/319.html). Our results could thus serve as a foundation for information on the characteristics of adolescents and thus provide school administrators and teachers with ideas to tailor activities to promote student’s positive strengths through those activities. Our results can also serve as a baseline for future school-based surveys in order to assess the effectiveness of this government initiative.

Our study has strengths in terms of the large sample size representing Thai adolescents of this school age and high response rate. As education is compulsory in Thailand up to year 9, most adolescents of this age group are in school, and therefore our sample well represented young adolescents in the whole country. However, there are a few limitations which should be acknowledged. Firstly, cross-sectional study designs preclude establishment of direction of causation between hypothesized explanatory variables and study outcomes. Secondly, recall bias and social desirability bias may also exist given the 12-month reference periods for risk behaviors and the inquiry about some sensitive questions such as drug use. Thirdly, although four common adolescent health-risk behavior categories were included in the analysis, some were not included, for example unhealthy diet and physical inactivity. Fourthly, although the response rate was very high - the sample included non-absentees and non-dropouts, the biasing effects of school-absenteeism, which is typically highly prevalent among the high-risk-behavior adolescents could not be avoided. Finally, by dichotomizing some of the manifest variables such as the number of standard drinks typically consumed during a drinking occasion and frequency of binge drinking, detailed information may have been missed. However, this did not affect the result of the clustering of risk behaviors using the latent class analysis.

## Conclusion

This study supports previous literature in reporting the heterogeneity of adolescents in school and, using a latent class regression analysis, expands our understanding by identifying subgroups of adolescents with multi-dimensional combinations of problem behaviors. With this sample, evidence was found of three classes of students: one having higher drug, alcohol and tobacco use and other health-risk behaviors, one with alcohol and tobacco use and other health-risk behaviors, and one almost without any of these behaviors. Our study in the Thai context of early middle school also confirms previous findings related to predictors of risky behaviors in early adolescents. For example, males, poor academic achievement, and students with families and peers who used drugs were at a higher risk. Accordingly, preventive programs targeting both families and schools are encouraged, as they are able to address all of these problems at the same time. The latent class analysis provides a modern person-centered approach to detect clusters of health-risk behaviors [12], and the results lay a basis for future research and practice in the prevention field of alcohol, drug use and health-risk behaviors among adolescents.

## Abbreviations

AIC: Akaike Information Criterion
BIC: Bayesian Information Criterion
RRR: relative risk ratio
GPA: Grade point average
LCA: Latent class analysis
SE: Standard error
SSABIC: Sample size adjusted Bayesian Information Criterion
